# Presynaptic toxicity of the AD susceptibility gene BIN1

**DOI:** 10.1002/alz.088949

**Published:** 2025-01-03

**Authors:** Pierre Dourlen, Erwan Lambert, Alejandra Freire‐Regatillo, Carla Gelle, Valentin Leclerc, Nicolas Barois, Xavier Hermant, Tommy Malfoi, Patrik Verstreken, Philippe Amouyel, Frank Lafont, Devrim Kilinc, Jean‐Charles Lambert

**Affiliations:** ^1^ Univ. Lille, Inserm, CHU Lille, Institut Pasteur de Lille, U1167‐RID‐AGE, DISTALZ, Lille France; ^2^ Univ. Lille, CNRS, Inserm, CHU Lille, Institut Pasteur de Lille, U1019‐UMR9017‐CIIL, Lille France; ^3^ Univ. Lille, CNRS, Inserm, CHU Lille, Institut Pasteur de Lille, US41‐UMS2014‐PLBS, Lille France; ^4^ Department of Neurosciences, Leuven Brain Institute, VIB Center for Brain & Disease Research, KU Leuven, Leuven Belgium

## Abstract

**Background:**

*BIN1* is a major susceptibility gene for AD and BIN1 protein interacts with Tau. However, the contribution of BIN1 and its isoforms to AD pathogenesis remains unclear. We recently described that human BIN1 isoform1 (BIN1iso1) induces an accumulation of early endosome vesicles leading to neurodegeneration in *Drosophila* retina and that the early endosome size regulation was conserved in human induced neurons. This role was specific to BIN1iso1, as compared to BIN1iso8 and BIN1iso9. Because endosomal trafficking is essential for synapse, we further analyzed BIN1 isoforms neurotoxicity at the synaptic level.

**Method:**

We first used electrophysiology, immunofluorescence and electron microscopy *in vivo* in transgenic *Drosophila* adult retina photoreceptor neurons and larval neuromuscular junction. We then tested a functional conservation in transduced rat primary neurons using tricompartment microfluidic devices, immunofluorescence and Multi‐Electrode‐Array to assess a presynaptic vs post‐synaptic role.

**Result:**

We observed an early loss of synaptic transmission upon BIN1iso1 expression specifically, in *Drosophila* retina photoreceptor neurons. This was characterized by a strong accumulation of abnormally large vesicles in the presynaptic compartment. In addition, expression of BIN1iso1 in motoneurons of the neuromuscular junction altered the morphology of synaptic boutons, with an increase in their number and a decrease in their size, and the appearance of satellite boutons. BIN1iso1 synaptic toxicity could be partially rescued by modulating endosomal regulators, suggesting that an endosomal defect would be the cause of synaptotoxicity. Finally, we observed a functional conservation in rat primary neurons as shown by the loss of synaptic connectivity only when expressing BIN1iso1 in the presynaptic compartment.

**Conclusion:**

Our results suggest that BIN1 has an isoform‐specific, deleterious effect on synaptic integrity when expressed in the presynaptic terminal. This effect could contribute to the synaptic loss observed early in AD.

